# Diagnostic Utility of Simple Inflammatory Markers for Metabolic Dysfunction-Associated Steatotic Liver Disease and Metabolic Syndrome in Children with Obesity

**DOI:** 10.3390/nu18142298

**Published:** 2026-07-14

**Authors:** Aleksandra M. Zajkowska-Sierpniak, Kamila A. Kwiatek-Średzińska, Monika Kowalczuk-Krystoń, Anna Lebensztejn, Anna Bobrus-Chociej, Marta Flisiak-Jackiewicz, Beata Cudowska, Dariusz M. Lebensztejn

**Affiliations:** 1 Department of Pediatrics, Gastroenterology, Hepatology, Nutrition, Allergology and Pulmonology, Medical University of Bialystok, Waszyngtona Street 17, 15-274 Bialystok, Poland; monika.kowalczuk-kryston@umb.edu.pl (M.K.-K.); anna.bobrus-chociej@umb.edu.pl (A.B.-C.); marta.flisiak-jackiewicz@umb.edu.pl (M.F.-J.); beata.cudowska@umb.edu.pl (B.C.); dariusz.lebensztejn@umb.edu.pl (D.M.L.); 2 Students’ Scientific Association Affiliated with the Department of Pediatrics, Gastroenterology, Hepatology, Nutrition, Allergology and Pulmonology, Medical University of Bialystok, Waszyngtona Street 17, 15-274 Bialystok, Poland; 41269@student.umb.edu.pl

**Keywords:** inflammatory markers, metabolic dysfunction-associated steatotic liver disease, metabolic syndrome, obesity, children

## Abstract

**Background:** Obesity markedly increases the risk of metabolic syndrome (MetS) and metabolic dysfunction-associated steatotic liver disease (MASLD), both of which are characterized by persistent low-grade inflammation. This study aimed to evaluate the diagnostic utility of complete blood count-derived inflammatory markers, including neutrophil-percentage-to-albumin ratio (NPAR), neutrophil-to-lymphocyte ratio (NLR), platelet-to-lymphocyte ratio (PLR), and lymphocyte-to-monocyte ratio (LMR), for MASLD and MetS in children with obesity. **Methods:** Children with obesity aged 7–18 years were prospectively enrolled and underwent anthropometric assessment, laboratory evaluation, and transient elastography with controlled attenuation parameter (CAP) measurement. Participants were classified into MASLD (CAP ≥ 250 dB/m) and non-MASLD groups. Children aged ≥10 years were additionally evaluated for MetS according to the International Diabetes Federation criteria. Logistic regression analyses assessed associations between inflammatory markers and MASLD or MetS, while receiver operating characteristic (ROC) analysis evaluated their diagnostic performance. **Results:** Children with MASLD had significantly higher NLR and lower LMR values than those without MASLD. Higher NLR (OR = 1.946, 95% CI: 1.092–3.467, *p* = 0.024) and lower LMR (OR = 0.722, 95% CI: 0.539–0.966, *p* = 0.028) were associated with MASLD, whereas NPAR and PLR were not. After adjustment for age, sex, body mass index and *C*-reactive protein, both NLR and LMR remained independently associated with MASLD. ROC analysis showed moderate diagnostic performance (AUC = 0.629 for NLR and 0.616 for LMR). None of the evaluated markers were associated with MetS. **Conclusions:** NLR and LMR were independently associated with MASLD in children with obesity and may represent complementary screening biomarkers for pediatric MASLD. However, given the cross-sectional design and moderate diagnostic performance observed in this study, prospective multicenter studies are needed to validate these findings before clinical implementation.

## 1. Introduction

The recent statistics available from the World Health Organization (WHO) indicate a concerning increase in the prevalence of pediatric obesity. In 1990, approximately 2% of children and adolescents aged 5–19 years were living with obesity. By 2022, this figure had risen to 8%, corresponding to more than 160 million affected children and adolescents worldwide [1]. More recent projections from the World Obesity Federation indicate that the number of children and adolescents aged 5–19 years living with obesity may increase from approximately 177 million in 2025 to 228 million by 2040, with the global prevalence rising from 8.7% to 11.9% over the same period [2].

Childhood obesity is a major contributor to early metabolic disturbances and is closely linked to both metabolic syndrome (MetS) and metabolic dysfunction-associated steatotic liver disease (MASLD) [3,4,5,6,7]. These disorders involve common biological processes including impaired insulin signaling, adipose tissue dysfunction and persistent low-grade inflammation. Chronic low-grade inflammation is recognized as a key mechanism underlying the relationship between obesity, MetS, and MASLD [7,8]. Visceral adiposity is particularly important, as it promotes immune–metabolic activation and the release of inflammatory mediators that may worsen insulin resistance and liver injury [8]. These abnormalities may already develop during childhood, before overt metabolic complications become clinically apparent. This provides a basis for investigating simple inflammatory indices as potential tools for early risk assessment and reducing the risk of long-term complications in children with obesity.

MASLD, previously termed non-alcoholic fatty liver disease (NAFLD), is a common liver disorder linked to metabolic abnormalities and may advance from hepatic fat accumulation and inflammation to fibrosis, cirrhosis, or hepatocellular carcinoma. Its frequency is particularly high among children and adolescents with obesity and is expected to rise in parallel with the increasing global burden of pediatric obesity. MASLD develops through interactions between genetic susceptibility, hormonal and lifestyle factors and metabolic abnormalities such as excess adiposity, dyslipidemia, insulin resistance, and impaired glucose metabolism [9,10,11,12,13,14,15,16]. Dietary factors also play a central role in the development, prevention, and management of pediatric MASLD. Excessive intake of energy-dense foods, saturated fats, ultra-processed products, and sugar-sweetened beverages, particularly those rich in fructose, may promote hepatic fat accumulation, insulin resistance, and low-grade inflammation. Conversely, improvement in diet quality, reduction in excessive caloric intake, and lifestyle modification remain key elements in the prevention and management of MASLD in children and adolescents [17]. Although MASLD represents an important health concern in children, its identification remains challenging because reliable, validated, and readily available diagnostic methods are still limited [5,16].

MetS comprises a group of coexisting metabolic disorders, such as elevated blood pressure, abnormal lipid profile, impaired insulin sensitivity and visceral adiposity. This condition markedly contributes to the development of cardiovascular complications and type 2 diabetes mellitus making early identification and appropriate management a clinical priority [18,19].

Reliable biomarkers capable of identifying and monitoring inflammatory processes are needed. Inflammatory indices calculated from routine complete blood count parameters, including neutrophil percentage-to-albumin ratio (NPAR), neutrophil-to-lymphocyte ratio (NLR), platelet-to-lymphocyte ratio (PLR), and lymphocyte-to-monocyte ratio (LMR), provide insight into immune homeostasis and the general level of inflammation. Although NLR and other inflammatory biomarkers have been widely studied in adults with MASLD, evidence in pediatric populations remains scarce and inconsistent. Furthermore, only a limited number of studies have evaluated several routinely available inflammatory biomarkers simultaneously in relation to both MASLD and MetS in children with obesity. Therefore, further studies are needed to clarify their clinical utility in pediatric patients [20,21,22,23,24,25,26,27]. This study aimed to assess the associations between systemic immune–inflammatory biomarkers (NPAR, NLR, PLR, and LMR) and the presence of MASLD and MetS in children and adolescents with obesity, and to evaluate their potential value as supportive screening markers for these conditions.

## 2. Materials and Methods

This study included children with obesity who were hospitalized in the Department of Pediatrics, Gastroenterology, Hepatology, Nutrition, Allergology and Pulmonology of the Medical University of Bialystok, Poland, because of suspected liver disease requiring further diagnostic evaluation. The subsequent inclusion criteria were implemented: pediatric patients between the ages of 7 and 18, with obesity defined by age- and sex-specific body mass index (BMI) percentiles based on Polish 2010 reference standards [28] and suspected liver disease—hepatomegaly and/or raised alanine aminotransferase (ALT) activity and/or fatty liver in an ultrasound examination. The research adhered to the principles of the Declaration of Helsinki and received approval from the Ethics Committee of the Medical University of Bialystok (approval No. APK.002.373.2023). Written informed consent was obtained from all patients’ legal guardians before enrollment. All individuals underwent anthropometric, laboratory and liver assessment using transient elastography (FibroScan, Echosens, Paris, France) with controlled attenuation parameter (CAP), performed by an experienced operator consistent with the manufacturer’s instructions. Based on CAP measurements, hepatic steatosis was diagnosed according to established cut-off values (CAP ≥ 250 dB/m) [7]. The diagnosis of MASLD was established in accordance with the multisociety consensus statement [9]. All obese patients were categorized into two groups: those with hepatic steatosis (MASLD) and those without hepatic steatosis (non-MASLD). In addition, children aged ≥10 years were divided into two groups based on the presence of MetS. The diagnosis of MetS was established according to the criteria determined by the International Diabetes Federation (IDF) in 2007. Central obesity was required in conjunction with two or more of the following conditions: raised triglycerides (TG), reduced high-density lipoprotein (HDL) cholesterol, elevated blood pressure, raised fasting plasma glucose [19]. Participants with incomplete data, acute infectious disease within two weeks before evaluation, or other liver conditions, such as hepatitis C virus (HCV) or hepatitis B virus (HBV) infection, autoimmune hepatitis, selected genetic and metabolic liver diseases (Wilson’s disease, alpha-1-antitrypsin deficiency), celiac disease, cystic fibrosis, toxic- and drug-induced liver injury or other chronic systemic inflammatory diseases, patients treated with corticosteroids or immunosuppressive drugs, were excluded from the analysis.

The exposure variables of interest were systemic inflammatory markers derived from routine laboratory parameters, including NPAR, NLR, PLR, and LMR. These inflammatory indices were calculated as follows: NPAR was expressed as the ratio of neutrophil percentage to serum albumin concentration; NLR as the ratio of neutrophil count to lymphocyte count; PLR as the ratio of platelet count to lymphocyte count and LMR as the ratio of lymphocyte count to monocyte count. These markers were evaluated as potential non-invasive indicators of MASLD and MetS in children with obesity.

The following covariates were included: anthropometric, metabolic, liver function and inflammatory parameters potentially associated with MASLD and MetS. Anthropometric variables comprised BMI, waist and hip circumference, and waist-to-hip ratio (WHR). Metabolic covariates included routine parameters obtained from fasting blood samples, such as glucose, insulin, and lipid profile components. Liver function parameters included activity of ALT, aspartate aminotransferase (AST), gamma-glutamyl transferase (GGT), and bilirubin concentration. Inflammatory parameters comprised white blood cell (WBC) and platelet (PLT) count, as well as *C*-reactive protein (CRP) concentration. All covariates were selected on the basis of their clinical relevance and previously stated associations with MASLD and MetS in pediatric populations. Data were derived from patients for whom complete measurements were available. All laboratory tests were performed at the Department of Pediatric Laboratory Diagnostics. The complete blood count was assessed using a Hematology Analyzer (Beckman Coulter). Biochemical parameters were determined by standard laboratory methods (Cobas 6000-c501, Roche Diagnostics, Mannheim, Germany). Homeostatic model assessment for insulin resistance (HOMA-IR) was calculated using the standard formula.

Results are presented as median, minimum, and maximum values. Comparisons between MASLD and non-MASLD groups, as well as between MetS and non-MetS groups, were performed using the Mann–Whitney U test for non-parametric data. In order to account for multiple evaluations, the false discovery rate (FDR) correction by the Benjamini–Hochberg procedure was applied to comparisons between groups. Statistical significance was defined as a *p*-value of less than 0.05. The association between systemic inflammatory markers (NPAR, NLR, PLR, and LMR) and the presence of MASLD and MetS was evaluated using logistic regression analysis. Univariate logistic regression models were constructed for each inflammatory marker, with MASLD or MetS status as the dependent variable. Moreover, multivariate logistic regression models were created for inflammatory markers that showed statistical significance in univariate logistic regression analysis, adjusting for age, sex, BMI, and CRP concentration. The strength of associations was reported as odds ratios (ORs) with corresponding 95% confidence intervals (CIs). Receiver operating characteristic (ROC) curve analysis was performed to assess the diagnostic accuracy of inflammatory markers for identifying MASLD and MetS. ROC curves were generated for each marker, and the area under the curve (AUC) was estimated as a measure of discriminatory ability. Optimal cut-off values were determined by the Youden index. For each cut-off value, sensitivity and specificity were calculated to assess diagnostic performance. All statistical analyses were performed using Statistica 13.3 software.

## 3. Results

**Figure 1:**
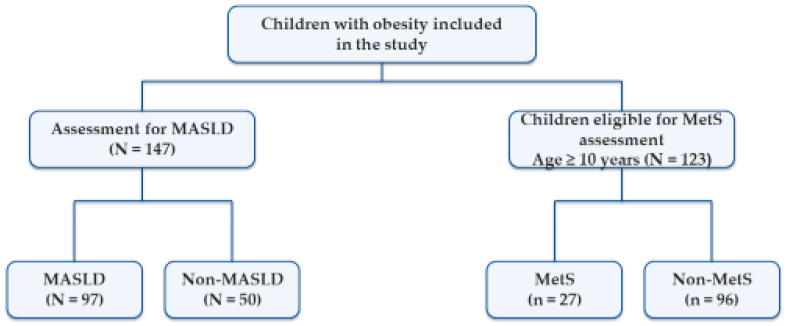
Flowchart of the study cohort.

A total of 147 children with obesity were included in the study, comprising 97 individuals (66%) with MASLD and 50 (34%) without MASLD. Among children aged ≥10 years (N = 123), 27 patients (22%) were classified as having MetS and 96 (78%) as non-MetS (Figure 1).

Comparative analysis demonstrated higher NLR values in the MASLD group compared with the non-MASLD patients (1.52 vs. 1.36) and significantly lower LMR values (3.89 vs. 4.47), whereas no significant differences were observed for NPAR and PLR. Children with MASLD also presented significantly higher anthropometric parameters, including BMI, waist and hip circumference, as well as higher insulin levels and HOMA-IR. Liver function parameters (ALT, AST, GGT) were significantly elevated in the MASLD group. Among conventional inflammatory markers, only WBC count was significantly higher in patients with MASLD, while CRP concentration and PLT count remained comparable (Table 1).

None of the investigated inflammatory markers (NPAR, NLR, LMR, PLR) differed between children with and without MetS. Patients with MetS had significantly lower HDL levels and higher TG concentrations. The other metabolic parameters, anthropometric variables, liver enzymes, and conventional inflammatory markers (WBC, CRP, and PLT) were comparable across the groups (Table 2).

In univariate logistic regression analysis, NLR was positively associated with the presence of MASLD (OR = 1.946; 95% CI 1.092–3.467; *p* = 0.024), while LMR showed an inverse association (OR = 0.722; 95% CI: 0.539–0.966; *p* = 0.028) (Table 3). For MetS, none of the evaluated inflammatory biomarkers demonstrated statistically significant association (Table 4). In multivariable logistic regression analysis, after adjustment for age, sex, BMI and CRP, higher NLR (OR = 1.971; 95% CI 1.085–3.580 *p* = 0.026) (Table 5), as well as lower LMR values (OR = 0.725; 95% CI: 0.537–0.980; *p* = 0.026) (Table 6), remained independently associated with MASLD. In both models, BMI was also identified as an independent predictor of MASLD, whereas age, sex, and CRP concentration were not associated with the condition.

**Figure 2:**
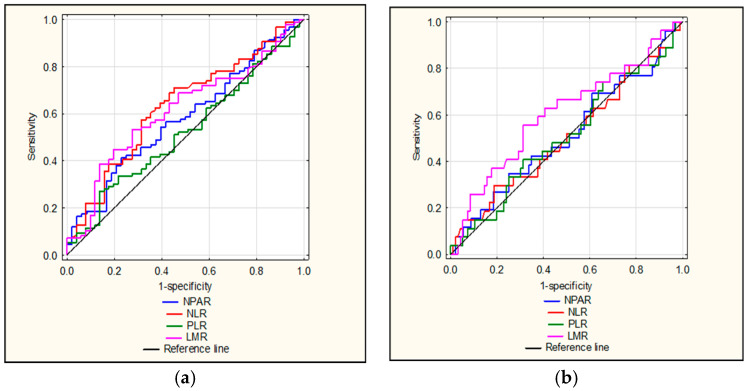
ROC curves presenting the diagnostic performance of systemic immune–inflammatory biomarkers (NPAR, NLR, PLR, and LMR) for identifying (**a**) MASLD and (**b**) MetS in children with obesity.

ROC analysis demonstrated moderate diagnostic performance of NLR (AUC = 0.629; 95% CI: 0.535–0.724; *p* = 0.007) and LMR (AUC = 0.616; 95% CI: 0.522–0.710; *p* = 0.016) for detecting MASLD. The optimal cut-off values were: ≥1.49 for NLR (sensitivity: 56.7%, specificity: 68%) and ≤3.94 for LMR (sensitivity: 53.6%, specificity: 72%) (Table 7, Figure 2a). In patients with MetS, ROC analysis showed limited diagnostic utility of the investigated inflammatory markers (Table 8, Figure 2b).

## 4. Discussion

This study evaluated the diagnostic utility of simple complete blood count-derived inflammatory indices for identifying MASLD and MetS in children with obesity. The main observation was that leukocyte-based ratios, particularly NLR and LMR, differed according to MASLD status, whereas no comparable association was found for MetS. These results suggest that pediatric MASLD may be accompanied by subtle systemic immune alterations, even when routine inflammatory markers show limited differences [21,22,23,27,29].

The pathophysiological relationship between obesity-related inflammation and pediatric MASLD is complex and multifactorial. Expansion of visceral adipose tissue promotes adipocyte dysfunction and macrophage infiltration, leading to increased secretion of pro-inflammatory cytokines, including interleukin-6 (IL-6) and tumor necrosis factor-alpha (TNF-α). These mediators contribute to insulin resistance, enhanced lipolysis, and increased delivery of free fatty acids to the liver, thereby promoting hepatic steatosis and hepatocellular injury. In addition, dysregulated adipokine secretion, including increased leptin activity and reduced adiponectin levels, may further contribute to hepatic inflammation and metabolic dysfunction. These mechanisms may explain why leukocyte-derived inflammatory indices, such as NLR and LMR, reflect inflammatory disturbances associated with MASLD in children with obesity [29,30,31,32].

Among the analyzed inflammatory biomarkers, leukocyte-based indices—particularly NLR and LMR—showed the strongest association with the presence of MASLD, whereas NPAR and PLR were not significantly connected with the condition. Pediatric patients with MASLD presented significantly higher NLR and lower LMR values, suggesting a shift in immune balance towards a pro-inflammatory response. This observation is consistent with previous studies reporting associations between leukocyte-derived indices and the severity of MASLD [21,22,23,24,27].

In the logistic regression analysis, NLR was positively related to MASLD, whereas higher LMR values were associated with lower odds of this condition. However, ROC analysis demonstrated only modest discriminatory ability with AUC values below the level expected for a strong diagnostic marker (NLR AUC = 0.629, LMR AUC = 0.616). Therefore, NLR and LMR should be interpreted as adjunctive indicators rather than diagnostic tests.

Importantly, the associations between NLR, LMR, and MASLD remained significant after adjustment for age, sex, BMI, and CRP concentration. In both multivariable models, BMI also remained an independent predictor, underscoring the relevance of obesity severity in the development of obesity-related metabolic complications, including MASLD. Nevertheless, the persistence of significant associations for NLR and LMR after adjustment for BMI indicate that the observed relationships were not solely explained by obesity and suggest that these indices may provide information beyond obesity severity alone. Taken together, these findings support their potential contribution to the early identification of children at increased risk of MASLD.

The present findings extend the existing literature by demonstrating that these associations are also present in a pediatric population with obesity. While previous studies have primarily focused on adult patients or evaluated single inflammatory markers, to our knowledge, the present study is one of the first to simultaneously assess four readily available systemic inflammatory indices in relation to both MASLD, and MetS. This approach provides additional evidence supporting the potential usefulness of leukocyte-derived inflammatory biomarkers in the early identification of pediatric MASLD. Furthermore, research on children may provide a more appropriate model of disease development and progression than studies on adults, because pediatric patients are less likely to have confounding factors, including comorbidities or medications taken.

Nevertheless, not all studies have demonstrated statistically significant differences. Duan et al. reported higher NLR and LMR values in the MASLD group compared with the simple obesity group. However, these differences did not achieve statistical significance [27].

Increased levels of inflammatory mediators and activated immune cells have been described in the progression of fatty liver disease, supporting the role of systemic inflammation in its pathogenesis. In contrast, the absence of significant differences for NPAR and PLR indicates that not all inflammatory markers derived from blood count parameters have equal diagnostic value in pediatric populations.

With regard to MetS, the associations between inflammatory biomarkers and disease presence were less pronounced. None of the investigated indices showed statistically significant relationships with MetS. This observation may reflect the complex and multifactorial nature of MetS, and suggest that leukocyte-based markers are more useful in identifying early liver-related inflammatory changes than generalized metabolic disorders [4,6,18,22].

An important aspect of this analysis was the comparison of the evaluated indices with conventional inflammatory markers, including WBC, PLT, and CRP. NLR and LMR were found to be more sensitive indicators of subtle inflammatory changes associated with MASLD than CRP and PLT. This may be attributed to the ability of ratio-based indices to better reflect the dynamic balance between different immune cell populations compared with single quantitative parameters [21,22,27].

In addition, the inflammatory markers were analyzed in relation to liver function parameters and metabolic indices. Significantly higher levels of liver enzyme activities (ALT, AST, GGT) were observed in children with MASLD, confirming the association between inflammatory processes and hepatocyte damage [14,16,29]. Moreover, patients with MASLD demonstrated higher insulin levels and HOMA-IR values, indicating a close association between this condition and metabolic disorders. These findings support the concept of MASLD as a hepatic manifestation of metabolic syndrome [6,7,13,18].

Nutritional factors are also highly relevant when interpreting the relationship between obesity, systemic inflammation, and MASLD in children. Excessive caloric intake, diets rich in saturated fat, and frequent consumption of sugar-sweetened beverages, particularly those containing fructose, may promote visceral adiposity, insulin resistance, and hepatic lipid accumulation. High fructose intake is especially important in pediatric populations because it may enhance de novo lipogenesis, increase intrahepatic fat deposition, and contribute to oxidative stress and low-grade inflammation. In this context, dietary habits may indirectly influence inflammatory biomarkers by contributing to obesity-related metabolic disturbances, insulin resistance, and hepatic steatosis. Therefore, lifestyle and nutritional interventions aimed at reducing excessive energy intake, saturated fat consumption, and fructose-rich beverages remain essential components of MASLD prevention and management in children with obesity [8,13,17,29].

The findings of this study may have several clinical implications. Since NLR and LMR are inexpensive, widely available, and derived from routine complete blood count parameters, they may facilitate identification of children with obesity who require closer metabolic and hepatological assessment. However, because their diagnostic performance was only moderate, these markers should not be used as standalone diagnostic tools for MASLD. Instead, they may serve as supportive indicators in combination with anthropometric, biochemical, and imaging-based assessments. This approach may be particularly useful in primary care or pediatric obesity clinics, where easily accessible tools are needed to support early risk stratification and referral decisions.

Several limitations of this study should be acknowledged. First, the relatively small sample size and the single-center design may limit the generalizability of the findings. In addition, although patients were evaluated according to a standardized protocol, the cross-sectional nature of the analysis does not allow causal relationships between inflammatory biomarkers and MASLD to be established. Therefore, the observed associations should be interpreted as correlational and hypothesis-generating rather than as evidence of a direct pathogenic role of these biomarkers. The study population was recruited from a single geographic region and was characterized by limited ethnic diversity, which may further restrict the applicability of the findings to broader pediatric populations. Moreover, magnetic resonance imaging-proton density fat fraction or liver biopsy were not performed to support MASLD diagnosis due to their limited availability and the need for general anesthesia. Another limitation is the lack of detailed dietary assessment. Data on dietary patterns, nutrient intake, fructose consumption, saturated fat intake, ultra-processed food consumption, and overall diet quality were not available in the present study. Therefore, we were unable to evaluate the potential influence of dietary factors on inflammatory biomarkers or MASLD, and future studies should include standardized dietary assessment tools to clarify the relationship between diet quality, systemic inflammation, and pediatric MASLD. Furthermore, NLR and LMR are nonspecific leukocyte-derived inflammatory indices and may be influenced by factors unrelated to MASLD, including subclinical infections, chronic inflammatory conditions, stress, or medication use. Although the multivariable models were adjusted for age, sex, BMI, and CRP concentration, the observed associations should be interpreted with caution. An additional limitation concerns the interpretation of NPAR, which includes serum albumin concentration. Albumin levels may be influenced not only by systemic inflammation but also by nutritional status, dietary protein intake, liver synthetic function, and protein loss. Because detailed nutritional assessment and dietary protein intake were not available, we could not fully evaluate the potential influence of nutritional status on albumin concentration and NPAR values. Finally, the diagnostic performance of the evaluated inflammatory markers was moderate and was observed only for MASLD, not for MetS.

Overall, the results suggest that NLR and LMR may represent potential supportive screening biomarkers for pediatric MASLD. However, given their moderate diagnostic performance and the cross-sectional design of the study, they should not be interpreted as standalone diagnostic tools. Further validation in prospective multicenter studies is required before their clinical application.

## 5. Conclusions

Childhood obesity is closely connected with metabolic complications such as MetS and MASLD, which remain important challenges in pediatric care [3,5,7,13,18]. From a clinical perspective, the results of the present study emphasize the importance of cautious interpretation of inflammatory biomarkers in children. Although NLR and LMR were associated with the presence of MASLD, their use in pediatric clinical practice is constrained by physiological age-related changes, the absence of validated age-specific reference thresholds and only moderate diagnostic accuracy. Therefore, NLR and LMR should not be considered standalone diagnostic tools, but rather potential supportive screening biomarkers that may complement existing anthropometric, biochemical and imaging-based assessments. Their accessibility, low cost, and availability from routine laboratory testing may support their role as adjunctive indices in the clinical assessment and risk stratification of pediatric MASLD [21,22,23,27]. These biomarkers may help identify children with obesity who require closer metabolic and hepatological evaluation. Further prospective multicenter studies are needed to validate these findings, establish standardized pediatric cut-off values and determine whether NLR and LMR can improve risk stratification and clinical decision-making in routine pediatric practice.

## Figures and Tables

**Table 1:** Comparison of selected anthropometric, metabolic, liver function and inflammatory parameters between MASLD and non-MASLD patients.

Parameter	MASLD Median (Range) N = 97	Non-MASLD Median (Range) N = 50	*p*	Adjusted *p*
NPAR	12.39 (8.51–19.14)	11.96 (7.02–17.13)	NS	NS
NLR	1.52 (0.75–5.22)	1.36 (0.62–3.30)	0.010	0.021
LMR	3.89 (1.30–8.06)	4.47 (2.25–9.13)	0.020	0.048
PLR	131.38 (75.88–330.85)	128.65 (68.88–203.53)	NS	NS
Anthropometric				
BMI (kg/m^2^)	29.75 (18.96–41.38)	26.41 (21.75–41.79)	<0.001	0.002
Waist circumference (cm)	100 (69–124)	91.5 (73–105)	<0.001	<0.001
Hip circumference (cm)	108 (69.5–131)	97 (82–127)	0.004	0.010
WHR	0.95 (0.82–1.07)	0.91 (0.78–1.06)	NS	NS
Metabolic, reference range				
Total cholesterol (mg/dL), 120–200	154 (88–239)	154.5 (104–261)	NS	NS
HDL (mg/dL), 35–65	45 (25–75)	46 (29–80)	NS	NS
LDL (mg/dL), <130	98 (50–180)	96.5 (43–186)	NS	NS
TG (mg/dL), 35–150	98 (34–336)	98 (39–306)	NS	NS
Glucose (mg/dL), 60–100	87 (71–109)	87.5 (74–104)	NS	NS
Insulin (μIU/mL), 2.6–24.9	22.16 (2.69–167.90)	14.12 (5.72–43.50)	<0.001	0.002
HOMA-IR	4.70 (0.52–35.2)	3.07 (1.19–9.55)	<0.001	0.003
Liver function, reference range				
ALT (U/L), <37	37 (11–174)	20 (10–72)	<0.001	<0.001
AST (U/L), <39	29 (12–123)	22 (10–43)	<0.001	<0.001
GGT (U/L), 9–40	21 (11–155)	15 (7–81)	<0.001	<0.001
Bilirubin (mg/dL), <1.2	0.46 (0.15–1.59)	0.47 (0.17–1.38)	NS	NS
Inflammatory, reference range				
WBC (×10^3^/μL), 4–10	7.22 (3.62–14.7)	6.58 (3.28–10.02)	0.017	0.038
CRP (mg/L), <5	1.58 (0.30–20.46)	1.27 (0.30–9.30)	NS	NS
PLT (×10^3^/μL), 140–450	304 (185–517)	295 (187–484)	NS	NS

N—number of cases, NS—nonsignificant, NPAR—neutrophil-percentage-to-albumin ratio, NLR—neutrophil-to-lymphocyte ratio, LMR—lymphocyte-to-monocyte ratio, PLR—platelet-to-lymphocyte ratio, BMI—body mass index, WHR—waist-to-hip ratio, HDL—high-density lipoprotein cholesterol, LDL—low-density lipoprotein cholesterol, TG—triglycerides, HOMA-IR—homeostasis model assessment of insulin resistance, ALT—alanine aminotransferase, AST—aspartate aminotransferase, GGT—gamma glutamyltransferase, WBC—white blood cells, CRP—*C*-reactive protein, PLT—platelets.

**Table 2:** Comparison of selected anthropometric, metabolic, liver function and inflammatory parameters between patients with and without MetS.

Parameter	MetS Median (Range) N = 27	Non-MetS Median (Range) N = 96	*p*	Adjusted *p*
NPAR	12.12 (8.87–17.13)	12.30 (7.02–17.66)	NS	NS
NLR	1.47 (0.75–4.74)	1.48 (0.62–5.22)	NS	NS
LMR	4.65 (2.24–7.30)	4.07 (1.30–9.13)	NS	NS
PLR	130.69 (68.88–208.33)	128.65 (76.01–330.85)	NS	NS
Anthropometric				
BMI (kg/m^2^)	31.83 (23.77–39.28)	29.01 (22.25–41.79)	NS	NS
Waist circumference (cm)	102 (80–123)	98 (73–124)	NS	NS
Hip circumference (cm)	109.5 (92–129)	107.25 (85–131)	NS	NS
WHR	0.95 (0.84–1.046)	0.94 (0.78–1.06)	NS	NS
Metabolic, reference range				
Total cholesterol (mg/dL), 120–200	166 (88–237)	153.5 (92–261)	NS	NS
HDL (mg/dL), 35–65	38 (29–65)	46 (25–80)	<0.001	<0.001
LDL (mg/dL), <130	97 (43–153)	95.5 (47–186)	NS	NS
TG (mg/dL), 35–150	180 (65–336)	93.5 (34–242)	<0.001	<0.001
Glucose (mg/dL), 60–100	87 (71–105)	87 (71–104)	NS	NS
Insulin (μIU/mL), 2.6–24.9	24.87 (5.72–50.32)	19.40 (6.03–167.90)	NS	NS
HOMA-IR	4.89 (1.19–10.40)	4.14 (1.37–35.20)	NS	NS
Liver function, reference range				
ALT (U/L), <37	30 (11–80)	28 (10–141)	NS	NS
AST (U/L), <39	24 (12–59)	25 (10–58)	NS	NS
GGT (U/L), 9–40	22 (10–87)	19 (7–65)	NS	NS
Bilirubin (mg/dL), <1.2	0.49 (0.15–1.29)	0.48 (0.20–1.59)	NS	NS
Inflammatory, reference range				
WBC (×10^3^/μL), 4–10	7.23 (3.62–10.51)	6.72 (3.28–12.90)	NS	NS
CRP (mg/L), <5	1.68 (0.44–9.48)	1.34 (0.30–20.46)	NS	NS
PLT (×10^3^/μL), 140–450	279 (187–517)	293 (185–484)	NS	NS

N—number of cases, NS—nonsignificant, NPAR—neutrophil-percentage-to-albumin ratio, NLR—neutrophil-to-lymphocyte ratio, LMR—lymphocyte-to-monocyte ratio, PLR—platelet-to-lymphocyte ratio, BMI—body mass index, WHR—waist-to-hip ratio, HDL—high-density lipoprotein cholesterol, LDL—low-density lipoprotein cholesterol, TG—triglycerides, HOMA-IR—homeostasis model assessment of insulin resistance, ALT—alanine aminotransferase, AST—aspartate aminotransferase, GGT—gamma glutamyltransferase, WBC—white blood cells, CRP—*C*-reactive protein, PLT—platelets.

**Table 3:** Univariate logistic regression analysis of inflammatory biomarkers for MASLD.

Parameter	OR (95% CI)	*p*
NPAR	1.178 (0.988–1.405)	NS
NLR	1.946 (1.092–3.467)	0.024
LMR	0.722 (0.539–0.966)	0.028
PLR	1.004 (0.995–1.014)	NS

OR—odds ratio, 95% CI—95% confidence interval, NPAR—neutrophil-percentage-to-albumin ratio, NLR—neutrophil-to-lymphocyte ratio, LMR—lymphocyte-to-monocyte ratio, PLR—platelet-to-lymphocyte ratio, NS—not significant.

**Table 4:** Univariate logistic regression analysis of inflammatory biomarkers for MetS.

Parameter	OR (95% CI)	*p*
NPAR	1.046 (0.845–1.295)	NS
NLR	1.200 (0.714–2.017)	NS
LMR	1.328 (0.957–1.841)	NS
PLR	0.999 (0.987–1.010)	NS

OR—odds ratio, 95% CI—95% confidence interval, NPAR—neutrophil-percentage-to-albumin ratio, NLR—neutrophil-to-lymphocyte ratio, LMR—lymphocyte-to-monocyte ratio, PLR—platelet-to-lymphocyte ratio, NS—not significant.

**Table 5:** Multivariate logistic regression model of NLR, adjusted for age, sex, BMI, and CRP concentration in MASLD.

Parameter	OR (95% CI)	*p*
NLR	1.971 (1.085–3.580)	0.026
Age	0.970 (0.819–1.148)	NS
Sex	1.421 (0.659–3.066)	NS
BMI	1.147 (1.024–1.284)	0.018
CRP	0.948 (0.799–1.124)	NS

OR—odds ratio, 95% CI—95% confidence interval, NLR—neutrophil-to-lymphocyte ratio, BMI—body mass index, CRP—*C*-reactive protein, NS—not significant.

**Table 6:** Multivariate logistic regression model of LMR, adjusted for age, sex, BMI, and CRP concentration in MASLD.

Parameter	OR (95% CI)	*p*
LMR	0.725 (0.537–0.980)	0.036
Age	0.991 (0.840–1.169)	NS
Sex	1.187 (0.558–2.527)	NS
BMI	1.140 (1.021–1.274)	0.020
CRP	0.967 (0.816–1.147)	NS

OR—odds ratio, 95% CI—95% confidence interval, LMR—lymphocyte-to-monocyte ratio, BMI—body mass index, CRP—*C*-reactive protein, NS—not significant.

**Table 7:** Diagnostic performance of inflammatory markers (NPAR, NLR, LMR, PLR) for identifying MASLD.

Parameter	AUC (95% CI)	Cut-Off	Se (%)	Sp (%)	PPV (%)	NPV (%)	LR+	LR−	DOR	*p*
NPAR	0.579 (0.482–0.677)	≥13.09	41.3	77.1	77.6	40.7	1.80	0.76	2.37	NS
NLR	0.629 (0.535–0.724)	≥1.49	56.7	68	77.5	44.7	1.77	0.64	2.78	0.007
LMR	0.616 (0.522–0.710)	≤3.94	53.6	72	78.8	44.4	1.92	0.64	2.97	0.016
PLR	0.523 (0.427–0.619)	≥157.56	27.1	86.3	78.8	38.6	1.97	0.85	2.32	NS

AUC—area under curve, 95% CI—confidence interval, Se—sensitivity, Sp—specificity, PPV—positive predictive value, NPV—negative predictive value, LR+—likelihood ratio positive, LR−—likelihood ratio negative, DOR—diagnostic odds ratio, NPAR—neutrophil-percentage-to-albumin ratio, NLR—neutrophil-to-lymphocyte ratio, LMR—lymphocyte-to-monocyte ratio, PLR—platelet-to-lymphocyte ratio, NS—not significant.

**Table 8:** Diagnostic performance of inflammatory markers (NPAR, NLR, LMR, PLR) for identifying MetS.

Parameter	AUC (95% CI)	Cut-Off	Se (%)	Sp (%)	PPV (%)	NPV (%)	LR+	LR−	DOR	*p*
NPAR	0.514 (0.383–0.646)	≥13.55	34.6	75	28.1	80.2	1.39	0.87	1.59	NS
NLR	0.508 (0.378–0.638)	≥2.10	29.6	81.3	30.8	80.4	1.58	0.87	1.82	NS
LMR	0.605 (0.478–0.732)	≥4.53	55.6	68.8	33.3	84.6	1.78	0.65	2.75	NS
PLR	0.510 (0.383–0.637)	≥15.78	40.7	68.8	26.8	80.5	1.30	0.86	1.51	NS

AUC—area under curve, 95% CI—confidence interval, Se—sensitivity, Sp—specificity, PPV—positive predictive value, NPV—negative predictive value, LR+—likelihood ratio positive, LR−—likelihood ratio negative, DOR—diagnostic odds ratio, NPAR—neutrophil-percentage-to-albumin ratio, NLR—neutrophil-to-lymphocyte ratio, LMR—lymphocyte-to-monocyte ratio, PLR—platelet-to-lymphocyte ratio, NS—not significant.

## Data Availability

The datasets generated and analyzed during the current study are available from the corresponding author on reasonable request due to patient confidentiality.

## Abbreviations

The following abbreviations are used in this manuscript:

WHO	World Health Organization
NPAR	Neutrophil-percentage-to-albumin ratio
NLR	Neutrophil-to-lymphocyte ratio
LMR	Lymphocyte-to-monocyte ratio
PLR	Platelet-to-lymphocyte ratio
BMI	Body mass index
WHR	Waist-to-hip ratio
HDL	High-density lipoprotein cholesterol
LDL	Low-density lipoprotein cholesterol
TG	Triglycerides
HOMA-IR	Homeostasis model assessment of insulin resistance
ALT	Alanine aminotransferase
AST	Aspartate aminotransferase
GGT	Gamma glutamyltransferase
WBC	White blood cells
CRP	*C*-reactive protein
PLT	Platelets
OR	Odds ratio
95% CI	95% confidence interval
AUC	Area under curve
Se	Sensitivity
Sp	Specificity
PPV	Positive predictive value,
NPV	Negative predictive value
LR+	Likelihood ratio positive
LR−	Likelihood ratio negative,
DOR	Diagnostic odds ratio
